# Individual- and environmental-related correlates of moderate-to-vigorous physical activity in 11-, 13-, and 15-year-old Finnish children

**DOI:** 10.1371/journal.pone.0234686

**Published:** 2020-06-18

**Authors:** Anette Mehtälä, Jari Villberg, Minna Blomqvist, Pertti Huotari, Timo Jaakkola, Pasi Koski, Taru Lintunen, Kaisu Mononen, Kwok Ng, Sanna Palomäki, Arja Sääkslahti, Tuija Tammelin, Tommi Vasankari, Sami Kokko

**Affiliations:** 1 Faculty of Sport and Health Sciences, University of Jyväskylä, Jyväskylä, Finland; 2 LIKES Research Centre for Physical Activity and Health, Jyväskylä, Finland; 3 KIHU – Research Institute for Olympic Sports, Jyväskylä, Finland; 4 Faculty of Education, University of Turku, Rauma, Finland; 5 School of Educational Sciences and Psychology, University of Eastern Finland, Joensuu, Finland; 6 Department of Physical Education and Sport Sciences, Physical Activity for Health Research Cluster, University of Limerick, Limerick, Ireland; 7 Urho Kaleva Kekkonen Institute for Health Promotion Research, Tampere, Finland; Teesside University/Qatar Metabolic Institute, UNITED KINGDOM

## Abstract

The objective of this study was to analyze the associations of various individual- and environmental-related factors with subgroups of daily, frequent, moderate and low moderate-to-vigorous physical activity (MVPA) among children and adolescents. Data were obtained from the Finnish School-age Physical Activity (FSPA) study 2016 from 4677 national representative 11-, 13-, and 15-year-old children and adolescents. MVPA and individual- and environmental-related factors were assessed by a questionnaire and analyzed by two-level logistic regression. Seventeen of the twenty-one variables were statistically significantly associated with MVPA. However, only three variables were statistically significant in all MVPA subgroups, whereby self-directed PA at least twice a week, fewer perceived barriers, and higher peer support increased the odds of participating in more MVPA. The results from this study showed essential differences among the MVPA subgroups, also supporting previous findings, whereby various individual- and environmental-based factors are associated with children and adolescents’ levels of MVPA. Challenges to designing and implementing effective interventions are based on the need to account for individual differences within the population, as well as the varied connections between PA with different social and physical environments where children and adolescents’ PA takes place. PA interventions with various actions at multiple levels are warranted.

## Introduction

Engaging in physical activity (PA) in childhood is positively related to short- and long-term effects on physical health and mental wellbeing and adulthood PA [1,2]. Being active at all PA intensity levels has favorable impact on health, but the most robust evidence for disease prevention is moderate-to-vigorous PA (MVPA) intensity level. Sufficient amounts of MVPA has consistently shown to be beneficial on cardio-metabolic risk factors, body composition, physical fitness, bone health, motor skills, psychological distress and well-being [1]. Despite this, four out of five children and adolescents worldwide are not meeting the PA recommendations that is, do not participate every day in at least 60 minutes of MVPA [3–5].

The cumulative results of prior research have shown PA is a complex phenomenon influenced by multiple biological, psychosocial, structural and cultural environmental factors [6–11]. Unsurprisingly, the integration of results indicates the challenges to present an unambiguous overview of PA correlates. Prior work has concentrated mainly on typical or usual values in heterogeneous data, combining measures obtained from physically inactive and active individuals [7,9–11]. Few studies have investigated factors related to compliance with PA recommendations [5,12–15]. Little is known about correlates in other levels of PA among children and adolescents.

To better understand the PA of children and adolescents, it is necessary to consider central child- and adolescent-specific variables and contexts that relate to their everyday PA [16]. The sufficient amount of PA, from the health point of view, is rarely obtained in one context, but accumulates during the whole day in different social and physical settings [17]. For children and adolescents, school is the one of the most important institutions they are involved in and where they spend a substantial amount of time. A large part of it is spent sitting or standing in lessons, so to ensure recommended levels of PA are reached, families, surrounding society and community settings such as sports clubs must be actively involved [18].

From the other point of view, besides educational classes, many other school-related factors influence children and adolescents’ PA, including the physical opportunities of recess and the socializing influence of peers [19]. Meeting the recommended levels of PA has been observed to be associated with active transportation to school [14] and with attendance at a larger school or a school in an urban location [13]. It has also been found to be associated with participation in an organized afterschool activity and in everyday physical education (PE) classes [15]. School also provides opportunities to participate in a wide range of physical activities for children and adolescents in lower income families, who might otherwise not be able to afford attendance in sports or hobbies [20]. Furthermore, because of the mandatory nature of school attendance in most countries, the schools can intervene in the behavior of the least active children and adolescents who are otherwise difficult to reach [21].

Within the socio-ecological model the individual’s characteristics interact with their sociocultural and physical environments [22]. The impact of the environment on individuals’ PA is depending on the domain (e.g. transport or leisure time) and the context (e.g. outdoors, team-based, alone, or with friends) where PA occurs [16]. Moreover, environmental factors do not influence PA behaviors in isolation, but interact synergistically at several levels of PA influence [10]. From the perspective of health promotion, it is important to identify children and adolescents who do not take part in recommended amounts of PA. Thus, this study examines which factors are associated with different levels of PA and are relevant for children and adolescent’s PA. The purpose of this study was to examine the associations of various individual- and environmental related variables with different MVPA categories in a representative Finnish-speaking sample of 11-, 13-, and 15-year-old children and adolescents within the socio-ecological model [9,22].

## Methods

### Participants

The current cross-sectional study is based on survey data gathered in 2016 from a national monitoring study; the Finnish School-age Physical Activity (FSPA). The goal of FSPA is to monitor children and adolescents’ PA and sedentary behavior, as well as to evaluate associated factors and time trends [23].

Children and adolescents were recruited randomly via selected primary and secondary schools. The schools were identified through a method based on a regional stratification in the capital area, southern, central, and northern Finland to complete a nationally representative sample. All school principals received an invitation letter with details of the background, objectives and practices of the study. Participation was voluntary, and schools could refuse to participate to the study or cancel their participation without any consequences. Written consent was not obtained. Of the Finnish-speaking schools, 285 (62%) agreed to participate to the FSPA-study. This included 7,916 children and adolescents aged 11 years (grade 5), 13 years (grade 7) and 15 years (grade 9).

Children, adolescents, and their parents were informed of the nature of the study. Participation was voluntary, and the pupils could refuse or stop to answer the questionnaire without any consequences at any time, i.e. passive consent protocol was used. Active consent was obtained only if municipalities or cities required it. Some municipalities and cities required active consent from parents or guardians of the participants, which was applied for and approved. Questionnaire was anonymous. The study protocol was approved by the local ethics committee 17.3.2014.

Students completed a teacher-administered online questionnaire in their classrooms during one lesson plus recess time, for a maximum of 60 min between March and May of 2016. A total of 4714 Finnish-speaking children and adolescents answered the FSPA 2016 questionnaire, with a response rate of 60%. The final sample of this study was composed of 4677 children and adolescents who returned usable data. Four percent of the participants were born in a country other than Finland, and among those countries, Estonian was the most commonly reported country of birth (1% of the total study sample).

### Physical activity

Participation in MVPA was measured by asking, “During the past 7 days, on how many days were you physically active for a total of at least 60 minutes per day?” [24]. The following introduction with the definition of MVPA was included before the question. “In the question, physical activity refers to all activities that increase your heart rate and makes you breath heavy some of the time, for example while exercising, while playing with friends, on the way to and from school, or during PE classes. Physical activities are, for example, running, brisk walking, roller skating, cycling, dancing, skateboarding, swimming, downhill skiing, cross-country skiing, soccer, basketball and baseball.” In this study MVPA was coded in four categories: 7 days as daily, 5–6 days as frequent, 3–4 days as moderate, and 0–2 days as low MVPA. The MVPA question has been widely used and has acceptable validity and reliability among adolescents. Intra-rater reliability from Finnish children and adolescents was moderate through the use of the same four MVPA categories as used in this paper (Kappa = 0.503) [25]. The usability of self-reported PA on surveillance studies has been supported by studies among adolescents and population subgroups [26,27].

### Independent variables

Twenty-one variables were included in the analysis and categorized according to Sallis and colleagues [9] as demographical and biological (4 variables), psychological, cognitive and emotional (4 variables), behavioral and skills (6 variables), social and cultural (2 variables) and physical environment (5 variables) variables (Table 1; groupings of response categories are presented in S1 Table). The internal consistencies of the items in the sum variables were acceptable and alpha values varied between .685 and .885 (S1 Table).

**Table 1:** Description of variables used in the analysis.

Variable	Response category	Mean (SD)	Proportion (%)
**Demographical and biological**			
Gender	Boy	N/A	48
	Girl	N/A	52
Grade	5^th^ (age 11)	N/A	36
	7^th^ (age 13)	N/A	35
	9^th^ (age 15)	N/A	29
Body mass index (BMI)	Thin/normal	18.8 (2.3)	86
	Overweight	24.6 (1.7)	11
	Obese	30.7 (3.2)	3
Family Affluence Scale (FAS) III	High	10.7 (0.9)	32
	Moderate	8.5 (0.5)	42
	Low	6.2 (1.1)	26
**Psychological, cognitive, and emotional**			
Perceived academic achievement	Good/very good	9.4 (0.5)	21
	Moderate/good	7.7 (0.5)	50
	Poor/moderate	5.4 (1.1)	29
Perceived physical competence	High	23.0 (1.4)	36
	Moderate	18.5 (1.1)	31
	Low	13.1 (2.8)	33
Perceived physical capacity	High	44.6 (2.7)	35
	Moderate	37.2 (2.0)	35
	Low	28.8 (4.2)	30
Perceived barriers	High	44.2 (10.)	33
	Moderate	58.3 (1.9)	34
	Low	63.8	33
**Behavioral attributes and skills**			
PA events (≤ 1 day/week)^a^			
School clubs	At least 2 days/ week	N/A	20
Other clubs (not sports clubs)	At least 2 days/ week	N/A	23
Private sports clubs	At least 2 days/ week	N/A	39
Sports clubs (non-profit)	At least 2 days/ week	N/A	39
Self-directed	At least 2 days/ week	N/A	75
Screen time (TV, PC, tablet, mobile, console games), ≤2h/day	0–2 days/week	N/A	30
	3–4 days/week	N/A	41
	5–7 days/week	N/A	29
**Social and cultural**			
Peer support for PA	High	16.2 (1.9)	30
	Moderate	11.6 (1.0)	35
	Low	6.5 (2.1)	35
Parental support for PA	High^b^	paternal: 30.6 (2.8) maternal: 30.1 (2.5)	38
	Moderate^c^	paternal: 23.0 (2.0) maternal: 23.5 (1.7)	23
	Low^d^	paternal: 14.0 (3.9) maternal: 15.4 (3.8)	39
**Physical environment**			
Recess	Outdoors	N/A	59
	Indoors	N/A	41
Residence	Urban	N/A	65
	Rural	N/A	35
School transportation, winter/spring and autumn	Active (walking, cycling)	N/A	52^e^/67^f^
	Non-active (motorized)	N/A	48^e^/33^f^
School policies and practices	High (10–15)	11.5 (1.5)	33
	Moderate (7–9)	7.9 (0.8)	40
	Low (1–6)	4.9 (1.2)	27

SD, standard deviation; N/A, Not applicable.

^a^row percentage of proportion that met the response category criteria

^b^parental support was categorized as high if both paternal and maternal support were high or one high and the other moderate

^c^parental support was categorized as moderate if both paternal and maternal support were moderate or one high and the other low

^d^parental support was categorized as low if both paternal and maternal support were low or one moderate and the other low

^e^school transportation in winter

^f^school transportation combined in spring and autumn

### Statistical analysis

Differences in the prevalence of genders and mean of age, body mass index (BMI) and Family Affluence Scale (FAS) of children and adolescents among the MVPA groups were assessed using chi-square tests and one-way ANOVA. Assumptions of a one-way ANOVA were checked using a range of graphical (e.g. histograms, quantile-quantile plots) and numerical methods. The age variable had a trimodal distribution, thus differences among groups were analyzed by using Kruskal-Wallis test. Statistical significance was determined at *p*-value of .05. Descriptive statistics were obtained using IBM SPSS Statistics for Windows, version 24 (IBM Corp., Armonk, N.Y., USA).

MVPA was divided to four MVPA subgroups: at least 60 minutes of MVPA in 7 days (yes/no), 5–6 days (yes/no), 3–4 days (yes/no), and 0–2 days (yes/no) per week. The associations of independent variables with MVPA subgroups were examined using two-level logistic regression on Stata version 15. Responses from children and adolescents (level 1) were nested within schools (level 2), and two-level logistic regression analysis was used to account for correlation among responses from within cluster-level. Logistic regression analyses were conducted on the overall sample individually for each of the four MVPA subgroups. Odds ratios (OR) and 95% confidence intervals (CI) were reported to indicate the association with MVPA. All variables (listed in Table 1) were selected in the models based on prior knowledge and on their possible associations with PA [6–11].

## Results

There were equal proportions of daily, frequently and moderately active children and adolescents (27–32%), whereas every eighth (12%) participant was assigned to the low active group (Table 2). Half (49%) of children and adolescents in the daily active group were from grade 5, whereas almost half (44%) of lower active groups were from grade 9. Mean body mass index (BMI) was higher in the lower MVPA groups. The percentages of overweight or obese children and adolescents were higher in lower MVPA groups than in higher MVPA groups, (χ^2^_6df_, = 61,14, p < .001). Mean Family Affluence Score (FAS) were highest in daily and frequent MVPA groups and lowest in the low MVPA group.

**Table 2:** Characteristics of the study participants by physical activity categories.

	Daily MVPA (n = 1354)	Frequent MVPA (n = 1482)	Moderate MVPA (n = 1290)	Low MVPA (n = 551)	All	N
Proportion (%)	29.0	31.7	27.6	11.8	100	4677
Gender (boy), (%)	58	46	40	48	48	4677
Age, mean (SD)	13.6 (1.6)	14.1 (1.6)	14.5 (1.6)	14.6 (1.7)	14.1 (1.7)	4667
5^th^ graders (%)	40	31	20	9	100	1664
7^th^ graders (%)	27	34	29	10	100	1629
9^th^ graders (%)	18	30	34	18	100	1366
BMI, mean (SD)	19.2 (3.2)	19.6 (3.2)	20.2 (3.5)	20.7 (4.1)	19.8 (3.4)	4464
Thin/normal weight (%)	30	33	26	11	100	3853
Overweight (%)	24	28	35	14	100	484
Obese (%)	23	20	29	28	100	118
FAS, mean (SD)	8.8 (2.0)	8.8 (1.8)	8.5 (1.8)	8.2 (2.0)	8.7 (1.9)	3991

SD, standard deviation; BMI, Body mass index; FAS, Family Affluence Scale III (range 0–13)

### Correlates in all MVPA subgroups

The results from the two-level logistic regression models containing all explanatory variables (full models) are in Table 3. Variables with statistically significant association with MVPA are emphasized and their odds (ORs) and 95% confidence intervals (CIs) in the Table 3.

Almost all variables included in the models were statistically significantly associated with MVPA. Moreover, perceived barriers to PA, self-directed PA, and peer support for PA were statistically significant in all MVPA subgroups, whereby the fewer perceived barriers, self-directed PA at least twice a week, and higher peer support had increased odds of daily or frequent MVPA. Participation in PA organized by the third sector, policies and practices of the school, and parental support for PA were statistically non-significant factors in this study.

**Table 3:** Two-level logistic regression analysis. Dependent variable MVPA participation in the previous 7 days.

	At least 60 minutes of moderate-to-vigorous physical activity (MVPA) per day for…in the last week.							
	7 days Daily MVPA		5–6 days Frequent MVPA		3–4 days Moderate MVPA		0–2 days Low MVPA	
	OR	95% CI	OR	95% CI	OR	95% CI	OR	95% CI
**Demographic and biological**								
Gender								
Boy	**1.57**	**1.25–1.96**	**0.81**	**0.67–0.99**	**0.78**	**0.62–0.97**	0.98	0.69–1.40
Girl	^a^1.0		^a^1.0		^a^1.0		^a^1.0	
Grade								
5^th^ (age 11)	**2.64**	**1.79–3.89**	0.81	0.60–1.11	**0.47**	**0.33–0.67**	1.40	0.81–2.40
7^th^ (age 13)	**2.00**	**1.45–2.77**	0.89	0.69–1.14	**0.74**	**0.56–0.97**	0.80	0.52–1.24
9^th^ (age 15)	^a^1.0		^a^1.0		^a^1.0		^a^1.0	
BMI								
Thin/Normal	0.95	0.44–2.05	1.14	0.58–2.24	1.45	0.72–2.92	**0.42**	**0.19–0.93**
Overweight	0.81	0.35–1.85	0.98	0.48–2.02	1.94	0.92–4.11	0.44	0.18–1.09
Obese	^a^1.0		^a^1.0		^a^1.0		^a^1.0	
FAS								
High	1.22	0.91–1.62	1.06	0.82–1.36	0.86	0.64–1.15	**0.60**	**0.38–0.97**
Moderate	0.91	0.69–1.19	1.06	0.84–1.34	1.06	0.82–1.37	0.88	0.59–1.30
Low	^a^1.0		^a^1.0		^a^1.0		^a^1.0	
**Psychological, cognitive, and emotional**								
Perceived academic achievement								
Good/very good	1.12	0.82–1.53	1.05	0.79–1.39	0.78	0.57–1.07	0.97	0.56–1.68
Moderate/good	0.78	0.60–1.02	1.25	0.99–1.58	0.81	0.63–1.03	**1.49**	**1.00–2.21**
Poor/moderate	^a^1.0		^a^1.0		^a^1.0		^a^1.0	
Perceived physical competence								
High	**1.79**	**1.18–2.70**	0.94	0.66–1.35	**0.58**	**0.39–0.87**	0.90	0.45–1.80
Moderate	0.91	0.63–1.30	1.34	1.00–1.78	0.93	0.69–1.25	0.86	0.53–1.38
Low	^a^1.0		^a^1.0		^a^1.0		^a^1.0	
Perceived physical capacity								
High	**1.81**	**1.17–2.81**	1.24	0.87–1.78	0.71	0.48–1.04	0.55	0.29–1.06
Moderate	**1.52**	**1.03–2.25**	1.21	0.90–1.64	0.93	0.69–1.25	0.65	0.41–1.01
Low	^a^1.0		^a^1.0		^a^1.0		^a^1.0	
Perceived barriers								
Low	**1.69**	**1.24–2.31**	1.30	0.99–1.71	**0.69**	**0.51–0.93**	**0.33**	**0.20–0.55**
Moderate	1.05	0.77–1.42	**1.49**	**1.16–1.92**	1.00	0.77–1.29	**0.49**	**0.33–0.73**
High	^a^1.0		^a^1.0		^a^1.0		^a^1.0	
**Behavioral attributes and skills**								
PA events								
School clubs	**0.69**	**0.52–0.91**	**1.29**	**1.01–1.64**	1.11	0.83–1.47	0.95	0.59–1.53
Other clubs	0.94	0.72–1.22	1.09	0.86–1.37	1.05	0.81–1.36	0.84	0.54–1.30
Private sports clubs	**1.34**	**1.06–1.68**	0.89	0.73–1.08	0.98	0.78–1.24	0.82	0.55–1.20
Sports clubs	**1.39**	**1.09–1.77**	1.01	0.82–1.26	**0.72**	**0.56–0.93**	0.87	0.56–1.37
Self-directed	**1.51**	**1.12–2.03**	**1.55**	**1.21–1.99**	**0.71**	**0.56–0.93**	**0.37**	**0.26–0.52**
PA occasions ≤ 1 day/week	^a^1.0		^a^1.0		^a^1.0		^a^1.0	
Screen time ≥ 2h/day								
0–2 days	**1.70**	**1.29–2.25**	0.79	0.61–1.01	**0.71**	**0.53–0.94**	1.27	0.82–1.96
3–4 days	1.05	0.81–1.37	1.01	0.81–1.27	0.95	0.74–1.22	1.06	0.70–1.58
5–7 days	^a^1.0		^a^1.0		^a^1.0		^a^1.0	
**Social and cultural**								
Peer support								
High	**1.70**	**1.18–2.45**	1.23	0.91–1.67	**0.69**	**0.50–0.94**	**0.53**	**0.32–0.86**
Moderate	1.09	0.76–1.57	**1.47**	**1.10–1.96**	0.87	0.65–1.15	0.73	0.49–1.10
Low	^a^1.0		^a^1.0		^a^1.0		^a^1.0	
Parental support								
High	1.06	0.79–1.42	1.15	0.90–1.48	0.84	0.63–1.11	0.83	0.52–1.33
Moderate	1.06	0.78–1.44	1.02	0.78–1.32	1.14	0.86–1.50	0.72	0.46–1.14
Low	^a^1.0		^a^1.0		^a^1.0		^a^1.0	
**Physical environment**								
Recess								
Outdoors	1.28	0.21–1.78	**0.74**	**0.57–0.96**	1.34	0.99–1.81	0.65	0.41–1.04
Indoors	^a^1.0		^a^1.0		^a^1.0		^a^1.0	
Residence								
Urban	0.87	0.67–1.13	0.84	0.68–1.05	**1.30**	**1.00–1.68**	1.30	0.88–1.92
Rural	^a^1.0		^a^1.0		^a^1.0		^a^1.0	
School transportation (winter)								
Non-active	0.95	0.72–1.26	1.00	0.79–1.27	1.05	0.80–1.39	1.31	0.83–2.08
Active	^a^1.0		^a^1.0		^a^1.0		^a^1.0	
School transportation (spring and autumn)								
Non-active	0.92	0.66–1.27	0.84	0.64–1.11	1.04	0.76–1.42	**1.64**	**1.01–2.67**
Active	^a^1.0		^a^1.0		^a^1.0		^a^1.0	
School policies and practices								
High	1.23	0.85–1.76	0.96	0.71–1.31	0.86	0.61–1.22	0.92	0.53–1.60
Moderate	1.16	0.84–1.59	1.06	0.82–1.38	0.83	0.62–1.11	0.98	0.62–1.54
Low	^a^1.0		^a^1.0		^a^1.0		^a^1.0	
**Random effects**								
School	**0.48**	**0.32–0.71**	0.20	0.06–0.65	0.39	0.24–0.63	0.28	0.05–1.62

OR, Odds Ratio; CI, Confidence Interval; BMI, Body mass index; FAS, Family Affluence Scale III; Bolded are statistically significant, p < 0.05.

^a^Reference group.

### Correlates of daily MVPA

Younger age groups and being a boy were associated with greater odds of daily MVPA. Age was the strongest predictor for this group. Additionally, high and moderate perceived physical capacity, high perceived physical competence, low level of screen time, as well as attending PA organized by private sports sector or sports clubs at least twice a week were associated with increased odds of daily MVPA. In contrast, attending PA organized by school clubs at least twice a week was associated with lower odds of daily MVPA.

### Correlates of frequent MVPA

Being a boy was associated with lower odds of frequent MVPA compared with being a girl. Also, spending most of the time or always outdoors at recess decreased the odds for frequent MVPA. Attending school PA clubs at least twice a week was associated with increased odds of frequent MVPA. Moderate perceived academic achievement, moderate perceived physical competence, low perceived barriers, and low screen time increased the odds for frequent MVPA, but their confidence intervals included or crossed the value 1 (*p* = .051–.059).

### Correlates of moderate MVPA

Being a boy was associated with lower odds of moderate MVPA. In this MVPA subgroup, both 11- and 13-year-olds had lower odds than 15-year-olds. The OR of high perceived physical competence compared with low was almost halved (OR 0.58, CI 0.39–0.87). There were greater OR for living in an urban environment compared with living in a rural one. The increased ORs of attending sports clubs fewer than twice a week and of a high level of screen time were also observed in this PA group.

### Correlates of low MVPA

Normal weight compared with being obese and high FAS compared with low FAS were associated with decreased odds of being low active. Perceived moderate academic achievement compared with poor academic achievement and non-active transporting to school in the spring and autumn were positively associated with low MVPA.

## Discussion

The aim of this study was to examine the associations of various individual and environmental factors to MVPA among children and adolescents in daily, frequent, moderate and low MVPA subgroups. One third of all the participants reported meeting the PA guidelines, which is in accordance with previous studies, where PA compliance rates were measured among Finnish samples through device-based measures [28] and by self-report questionnaires [29]. Youngest children were more likely to be physically active and have a healthy BMI. Boys reported daily MVPA more often than girls did. Yet, girls reported frequent and moderate MVPA more often than boys did. Based on the results of this study, special attention should be given to girls and as children get older. Although some levels of physical activity can contribute toward improvements in cardiometabolic health, particular emphasis is needed to promote meeting the PA recommendations of at least 60 minutes of MVPA on a daily basis [30]. There were no gender or age group differences in the low MVPA group, highlighting the importance of promoting PA for all children and adolescents. The present results suggest peer support for PA, promoting self-directed PA, as well as reducing the barriers (e.g. enable children and adolescents to design and use neighborhood PA facilities) faced by children and adolescents to be more physically active may increase children and adolescents’ MVPA.

Participation in PA organized by sports clubs or private sport sector on a regular basis, perceived physical capacity and perceived physical competence seemed to be of great importance in the daily MVPA group. The result is consistent with expectancy-value theory and recent research of development of children and adolescents’ physical competence [31–33]. Perceived competence appears to influence children and adolescents’ participation in PA or vice versa, whereby the higher the level of perceived competence, the more likely children and adolescents will engage in activity. Physical fitness is an outcome of regular participation in physical activity hence, children and adolescents in the daily MVPA group may have the highest level of physical fitness, and thus, the highest perceived physical capacity. Peer support and sports club participation are closely correlated to PA enjoyment, which in turn contributes significantly to MVPA [34]. Moreover, positive support from significant others and feelings of success enhance perceived physical competence. Children and adolescents who report their PA competence more positively are also more likely to continue their PA over time [33].

Ongoing discussions in the field concerning screen time and its relationship with physical (in)activity or sedentary behavior continue. In their review, Biddle and colleagues [7] observed screen time to be independent of PA or only slightly associated with activity on a daily or weekly basis. The results from our study indicated children and adolescents who had the least amount of screen time were in favor of participating in daily activity and vice versa. This is in line with the findings of Gomez and colleagues [35], who studied children and adolescent’s compliance rates with MVPA using data from 12 countries. Choices and use of screen-based devices of the different kind are increasing, hence increasingly competing with other leisure-time activities. Moreover, limiting screen time has become more complicated. Based on the results of this study we cannot assess the importance of family restrictions on screen time. One explanation for the results of our study can be that the participation in organized PA counteracts excessive time in front of a screen [36]. Nevertheless, the results of this study support the inclusion of screen time recommendations in PA guidelines.

In general, school-related factors played a bigger role in the frequent MVPA group than they did in the other MVPA subgroups. Recess spent always or mostly indoors was associated with frequent PA. In previous studies, time spent outdoors has been consistently associated with higher PA [7,9,10]. The result can reflect the impact of the national Finnish Schools on the Move (FSM) program, whereby one of the goals was to make indoor time at school, other than PE, more physically active [37]. Another reason for the discrepancy may be that, in the frequent MVPA group, the odds of being a girl was slightly increased (23%), and girls tend to prefer indoor activities to spending time outdoors [38].

Whereas sports clubs seem to contribute to increasing children and adolescents’ PA to the recommended levels, they are not suitable for all. Children and adolescents may lack physical competence as observed in the moderate MVPA subgroup. There is the central role of peers in perceived competence and in perception of sports to be fun and enjoyable [39,40]. Thus, efforts should be put into promoting a supportive and approving social environment in sports clubs. Sports clubs could make a difference in the battle against inactivity, because they create an environment that promotes not only physical activity levels but also social and mental well-being of children and adolescents [41].

Further, notwithstanding the benefits of participation in organized sport, the fees to the organized sports can be too high for the children and adolescents living in low-affluence households, as in the low MVPA group. Low levels of self-directed PA were strongly associated with a low frequency of 60 min of MVPA per day, and proved to be a statistically significant factor in each MVPA subgroup. The odds of the motorized transportation to school in the autumn and spring and obesity were significantly increased within this subgroup of low MVPA. Therefore, free or low-cost neighborhood sport or recreational facilities with sidewalk networks near the homes and schools of children and adolescent could be used in enhancing PA in the low MVPA subgroup. Substantial amount of research showing the health benefits from active transportation exists. Active transport in children and adolescents has been observed to predict higher PA in adulthood, and associate negatively with weight status [42]. Thus, the results of this and previous studies suggest urban planners should be involved in PA promotion. However, to inform land-use planning in detail, there must be more research, particularly through strategies that take into account children and adolescents’ preferences, interests and desires [4,22,43,44].

Surprisingly, parental support for PA proved to be a non-significant variable in every MVPA subgroup. There is a possibility the combination of maternal and paternal support for PA in a single index hindered the gender-based interaction observed in other studies [39,40]. Girls get more support for PA from their mothers and boys more support from their fathers [40]. Yet, boys received more support for PA from their parents than girls, and that may be a result from boys generally being more active and participate more in sports than girls [39]. Furthermore, parental support may be more relevant to children than to adolescents, who have more freedom to control their lives [45]. In addition to children and adolescent’s age, the strength of influence may vary with what kind of support (direct or indirect) is provided by parents. Peer support may be more important than parental support at the age groups of this study sample, but parents may still have a strong effect, especially on younger children’s PA [39]. The main message is that interventions for enhancing children and adolescents’ PA should look at tools to increase parents’ awareness of the importance of peers in fostering children and adolescents’ interest in PA. Intervention developers should help parents to identify and create opportunities for activities that their children enjoy and are able to participate in with their friends [46].

Whereas several individual variables (ranging from demographic-biological to behavioral correlates) were strongly associated with MVPA, only a few of the physical environmental-level variables were significant correlates to low, moderate and frequent MVPA and none to daily MVPA. This is in line with the previous studies, where individual-level factors are reported to play a larger role in PA than the environment where PA takes place [14]. However, we should bear in mind that, in this study, taking part in MVPA is considered as accumulation of PA during the entire day within different contexts. The school policies and practices variable may have had more explanatory power if we had focused on MVPA during school time. In addition, because of the reliance on self-reported data, only three items from the question battery were chosen to format the present school environment index. The items that were chosen to describe a relationship to PA itself or actions, rather than to circumstances or acts of PA promotion at school. In future studies, these issues should be considered using device-based measures.

### Limitations and future studies

In this study, we used self-reported data, which has many advantages, but it also has its limitations. Participation in PA was potentially overestimated when compared with a device-based measure [47]. We can assume any overestimation was at the same level among MVPA subgroups, allowing comparisons among the groups. Any self-report information may be incorrect despite the best efforts of the respondents to be honest and accurate and some caution is necessary when interpreting the conclusions.

The choice of the cut-points for MVPA subgroups was made and is justified in relation to the global PA recommendations of at least 60 minutes of MVPA on a daily basis [1], and there is evidence to support this recommendation [30]. It must be emphasized, the decision to group children and adolescents based on how many days they reported at least 60 min MVPA, allows individuals, who may accumulate MVPA with different amounts *at a week level*, to be categorized to the same MVPA subgroup.

From the socio-ecological point of view, it is essential to assess whether children and adolescents really perceive the ability to avail PA opportunities [8]. For this, self-reports are more suitable than device-based measures. Nonetheless, future studies should measure the environment both in multiple ways, combining the evaluation how much children and adolescents’ perceive their environment and device-based measures may differ as well as influence their PA [44]. In addition, because of its rapidly developing and ever-changing nature, there should be further examination of the relationships between screen time and PA.

Finally, the study population was representative of Finnish-speaking children and adolescents, but any generalization to cultures other than Finnish-speaking ones should be done carefully. Finnish is the native language of 88% of the Finnish population [48]. Additional studies are needed to expand our understanding of children and adolescents from different cultures and ethnic backgrounds, but also of children and adolescents with special educational needs [49,50].

## Conclusion

The results of this study confirm PA is a multifactorial behavior influenced by multiple demographical, psychosocial, behavioral, and environmental factors [9]. By addressing the factors identified in this study in health promotion, information can be used for targeting interventions to reach the children and adolescents who are not considered to be physically active enough. The results showed different amounts of MVPA were predicted by various variables. Whereas, participation regularly in PA organized by sports clubs plays a big role in reaching the recommended levels of PA, it seems self-directed PA has an even bigger role in promotion of PA of all children and adolescents.

Thus, a major challenge in the design and implementation of effective interventions is the ability to take into account the individuality (i.e. the subgroups of the populations) and varied connections between different levels of physical activity, as well as the social and physical environmental factors. It is essential to provide versatile and accessible PA facilities for children and adolescents [8], and focus on creating supportive and encouraging environments especially for the least physically active. Developing transport infrastructures that attract children and adolescents to take part in active transport, so PA would become an integral part of the daily activities, could enhance PA of the least physically active children and adolescents. Facilitating PA support of peers may be a key to overcome the PA barriers those many of children and adolescents perceive.

## Supporting information

S1 Table Description of variables used in the analysis. (DOCX)
