# Supplementary material for: Individual- and environmental-related correlates of moderate-to-vigorous physical activity in 11-, 13-, and 15-year-old Finnish children
Source: PLoS One. 2020 Jun 18;15(6):e0234686. doi: 10.1371/journal.pone.0234686 (PMC7302665; doi:10.1371/journal.pone.0234686)
Supplement: S1 Table — (DOCX) [file pone.0234686.s001.docx]

| S1 Table. *Description of variables used in the analysis* | | | | | | |
| --- | --- | --- | --- | --- | --- | --- |
| Variable *(resource)* | Description | Response category (range) | Mean (SD) | | Proportion (%) | |
| **Demographical and biological** |  | | | | | |
| Gender | Gender. Response options: Boy=1, Girl=2 | Boy | | N/A | | 48 |
|  |  | Girl | | N/A | | 52 |
| Grade | What grade are you in? Fifth=1, Seventh=2, Ninth=3 | 5^th^ | | N/A | | 36 |
|  |  | 7^th^ | | N/A | | 35 |
|  |  | 9^th^ | | N/A | | 29 |
| Body mass index (BMI) [1,2] | Self-reported age, gender height (cm) and weight (kg)  Age and gender adjusted kg/m2 grouping [1,2] | Thin/normal | | 18.8 (2.3) | | 86 |
|  |  | Overweight | | 24.6 (1.7) | | 11 |
|  |  | Obese | | 30.7 (3.2) | | 3 |
| Family Affluence Scale III (FAS) [3] | Sum of the following questions:  1. Does your family own a car (a passenger car, a delivery van, or a truck)? (No=0, Yes, one=1, Yes, two or more=2).  2. Do you have your own bedroom? (No=0, Yes=1).  3. How many computers (including laptops and tablets, not including game consoles and smartphones) does your family own? (None=0, One=1, Two=2, More than two=3).  4. How many bathrooms are there in your home? (None=0, One=1, Two=2, More than two=3).  5. Does your family have a dishwasher? (No=0, Yes=1).  6. How many times did you and your family travel abroad for holiday/vacation last year?  (Never=0, Once=1; Twice=2, More than twice=3).  *Cronbach's α = 0.685* | High (10–13) | | 10.7 (0.9) | | 32 |
|  |  | Moderate (8–9) | | 8.5 (0.5) | | 42 |
|  |  | Low (0–7) | | 6.2 (1.1) | | 26 |
| **Psychological, cognitive, and emotional** |  | | | | | |
| Perceived academic achievement [4] | Sum of the following questions:  How do you think you perform in… mathematics/first language….compared to others of the same age?  Response options: Very poorly=1, Poorly=2, Moderately=3, Good=4, Very good=5  *Cronbach's α=0.872* | Good/very good (9–10) | 9.4 (0.5) | | 21 | |
|  |  | Moderate/good (7–8) | 7.7 (0.5) | | 50 | |
|  |  | Poor/moderate (2–6) | 5.4 (1.1) | | 29 | |
| Perceived physical competence  *(Physical Self-Perception Profile)* [5] | Sum of the following questions:  What am I like?   1. I'm good at sports – I am bad at sports 2. I’m among the best when it comes to athletic ability - I’m not among the best when it comes to athletic ability 3. I am confident in sporting situations – I am not confident in sporting situations 4. I belong among the most competent when it comes to selecting students to sports activities – I don’t belong among the most competent when it comes to selecting students to sports activities (competitions, games etc.) 5. I am one of the first when it comes to the opportunity to perform the sporting tasks – I am hanging back when it comes to the opportunity to perform the sporting tasks   Each of the five items was rated on a five-point Osgood scale from 1 to 5. Scales were reversed prior to summation.  *Cronbach's α=0.819* | High (21–25) | 23.0 (1.4) | | 36 | |
|  |  | Moderate (17–20) | 18.5 (1.1) | | 31 | |
|  |  | Low (4–16) | 13.1 (2.8) | | 33 | |
| Perceived physical capacity [6,7] | Sum of the following questions:  What are you like in sports at school and during free time?   1. I have good endurance – I fatigue easily 2. I am fast – I am slow 3. I am strong – I am week 4. I have good flexibility – I am stiff 5. I have good balance – I don’t have good balance 6. I am skilled in handling the ball – I am poor in handling the ball 7. I am good at running and jumping – I am poor at running and jumping 8. I am skillful in sports and games – I am not skillful in sports and games 9. I can develop my physical performance – I can’t develop my physical performance 10. I want to develop my physical performance – I don’t want to develop my physical performance   Each of the ten items was rated on a five-point Osgood scale from 1 to 5. Scales were reversed prior to summation.  *Cronbach's α=0.769* | High (41–50) | 44.6 (2.7) | | 35 | |
|  |  | Moderate (34– 40) | 37.2 (2.0) | | 35 | |
|  |  | Low (8–33) | 28.8 (4.2) | | 30 | |
| Barriers [8,9] | Sum of the following questions:  Please rate the extent to which the following factors prevents your participation in sports and physical activity?   1. My home is not in the vicinity of a guidance of an interesting physical activity 2. Participation in sports is too expensive 3. My friends do not practice physical activity either 4. I'm not the athletic type 5. My health restricts participation in physical activities 6. I think physical activity is important, but I just don’t bother participate in physical activity 7. There is no time for sports 8. PE at school does not inspire me 9. Sweating during physical activity feels disgusting 10. There are no benefits in physical activity for me 11. Sports is boring 12. Sports is too competitive 13. Other   Response options: To a very large extent=1, To a large extent=2, To a moderate extent=3, To a small extent=4, Not at all=5.  *Cronbach's α=0.756* | Low (62–65) | 63.8 (1.2) | | 33 | |
|  |  | Moderate (55– 61) | 58.3 (1.9) | | 34 | |
|  |  | High (1–54) | 44.2 (10.0) | | 33 | |
| **Behavioral attributes and skills** |  | | | | | |
| PA events *(The Adolescent Health and Lifestyle survey)* | How often are you physically active in your free time…?  Response options: Less often than once a week or never, Once a week, 2–3 days per week, 4–5 days per week, 6–7 days per week |  |  | |  | |
|  | …at school clubs | At least 2 days/ week | N/A | | 20 | |
|  | ...at other clubs (not sports clubs) | At least 2 days/ week | N/A | | 23 | |
|  | …at private sports clubs | At least 2 days/ week | N/A | | 39 | |
|  | …at sports clubs (non-profit) | At least 2 days/ week | N/A | | 39 | |
|  | …self-directed | At least 2 days/ week | N/A | | 75 | |

| Screen time *(Kids out! survey)* | Think of the usual week. How many days a week do you spend looking at screens (TV, PC, tablet, mobile, console games) more than two hours a day?  Response options: 0, 1, 2, 3, 4, 5, 6, 7 days | 0–2 days/week | N/A | 30 |
| --- | --- | --- | --- | --- |
|  |  | 3–4 days/week | N/A | 41 |
|  |  | 5–7 days/week | N/A | 29 |
| **Social and cultural** |  |  |  |  |
| Peer support [10] | Sum of the following questions:  During the typical week: How often your peers   1. participate in physical activity or sports with you 2. ask you to participate in physical activity or sports 3. discuss with you about physical activity or sports 4. encourage you to be physically active or praise your sports performances   Response options: Never=1, Rarely=2, Sometimes=3, Often=4, Very often=5  *Cronbach's α=0.828* | High (14–20) | 16.2 (1.9) | 30 |
|  |  | Moderate (10–13) | 11.6 (1.0) | 35 |
|  |  | Low (1–9) | 6.5 (2.1) | 35 |
| Parental support [11] | Sum of the following questions:  How often does your mother/father (or your stepmother/-father, if your mother/father does not live in your primary home) usually…   1. participates in physical activity or sports 2. encourages you to participate in physical activity or sports 3. provides a ride to a sporting place or sports hobby 4. participates in physical activity or sports with you 5. watches your training, your games or your competitions 6. discusses about physical activity or sports with you 7. pays fees related to physical activity or sports   Response options: Never=1, Rarely=2, Sometimes=3, Often=4, Very often=5  *Cronbach's α (paternal)= 0.885, Cronbach's α (maternal)= 0.777* | High^a^  (father: 27–35, mother: 27–35) | paternal: 30.6 (2.8)  maternal: 30.1 (2.5) | 38 |
|  |  | Moderate^b^  (father: 20–26, mother: 21–26) | paternal: 23.0 (2.0)  maternal:23.5 (1.7) | 23 |
|  |  | Low^c^  (father: 1–19,  mother: 1–20) | paternal: 14.0 (3.9)  maternal: 15.4 (3.8) | 39 |
| **Physical environment** |  |  |  |  |
| Recess  *(Finnish Schools on the Move survey)* | Where do you usually spend recesses?  Response options: Always outdoors, Mostly outside, but sometimes inside, Mostly inside, but sometimes outside, Always indoors  Outdoors = mostly or always outdoors  Indoors = mostly or always indoors | Outdoors | N/A | 59 |
|  |  | Indoors | N/A | 41 |
| Residence  *[Health Behavior of School Children, (HBSC) survey]* | What kind of place do you live in?  Response options:  City; in the centre, City; outside the centre, Countryside; in the village centre, Countryside; outside the village centre  Urban = in the city centre or outside the centre  Rural = in the village centre or outside the village centre | Urban | N/A | 65 |
|  |  | Rural | N/A | 35 |
| School transportation, winter/  spring and autumn  *(Finnish Schools on the Move survey)* | How do you usually commute to school?  Response options: Walk, Cycle, driven in by parents, School transportation, Other motorized vehicle | Active  (walking, cycling) | N/A | 52^d^/67^e^ |
|  |  | Non-Active (motorized) | N/A | 48^d^/33^e^ |
| School policies and practices  *(Finnish Schools on the Move survey)* | Sum of the following questions:  Which of the response options best describes the current situation at your school?   1. We mainly go out at recess 2. Prolonged sitting periods are cut during the lessons 3. The school has clubs containing physical activities There are physical activity clubs at the school.   Response options: Never=1, Seldom=2, Sometimes=3, Often=4, Always=5  *Cronbach's α = 0.794* | High (10–15) | 11.5 (1.5) | 33 |
|  |  | Moderate (7–9) | 7.9 (0.8) | 40 |
|  |  | Low (1–6) | 4.9 (1.2) | 27 |
| Notes. SD, standard deviation; N/A, Not applicable  ^a^parental support was categorized as high if both paternal and maternal support were high or one high and the other moderate  ^b^parental support was categorized as moderate if both paternal and maternal support were moderate or one high and the other low  ^c^parental support were categorized as low if both paternal and maternal support were low or one moderate and the other low  ^d^school transportation in winter  ^e^school transportation combined in spring and autumn | | | | |

**References**

1. Cole TJ, Lobstein T. Extended international (IOTF) body mass index cut-offs for thinness, overweight and obesity. Pediatr Obes. 2012;7: 284–294. doi: 10.1111/j.2047-6310.2012.00064.x
2. Heinz A. SPSS Code for the "IOTF Body Mass Index Cut-Offs for Thinness, Overweight and Obesity in Children" by Cole & Lobstein (2012); 2017. Available from http://hdl.handle.net/10993/31448.
3. Hartley JEK, Levin K, Currie C. A new version of the HBSC Family Affluence Scale—FAS III: Scottish Qualitative Findings from the International FAS Development Study. Child Indic Res. 2016;9: 233–245. doi: 10.1007/s12187-015-9325-3
4. Eccles J, Wigfield A, Harold RD, Blumenfeld P. Age and Gender Differences in Children's Self- and Task Perceptions during Elementary School. Child Dev. 1993;64: 830-838.
5. Fox KR, Corbin CB. The Physical Self-Perception Profile: Development and preliminary validation. J Sport Exercise Psy. 1989;11(4): 408–430.
6. Jokinen J, Järvelä S. Oppilas oman fyysisen toimintakykynsä arvioijana [Master's thesis]. University of Jyväskylä; 2013. 72 p. Available from https://jyx.jyu.fi/handle/123456789/41911.
7. Lintunen T. Perceived Physical Competence Scale for children. Scand J Med Sci Sports.1987;9(1): 57–64.
8. Borisova L, Podalko E. Toward classification of motives. Int Rev Sociol Sport. 1975;10(3‒4): 45–62.
9. Nupponen H, Penttinen S, Pehkonen M, Kalari J, Palosaari A-M. The impact of school education study: Starting points for the study, methods and description data. [Koululiikunnan vaikuttavuus –tutkimus: Lähtökohdat, menetelmät ja aineiston kuvaus.] 2010. Rauma: Publications of University of Turku.
10. Sallis JF, Grossman RM, Pinski RB, Patterson TL, Nader PR. The development of scales to measure social support for diet and exercise behaviors. Prev Med. 1987;16: 825–836.
11. Prochaska JJ, Rodgers MW, Sallis JF. Association of Parent and Peer Support with Adolescent Physical Activity. Res Q Exerc Sport. 2002;73(2): 206–210. doi: 10.1080/02701367.2002.10609010
